# Highly conformal electrodeposition of copolymer electrolytes into titania nanotubes for 3D Li-ion batteries

**DOI:** 10.1186/1556-276X-7-349

**Published:** 2012-06-27

**Authors:** Nareerat Plylahan, Nana Amponsah Kyeremateng, Marielle Eyraud, Frederic Dumur, Hervé Martinez, Lionel Santinacci, Philippe Knauth, Thierry Djenizian

**Affiliations:** 1 Aix Marseille Université, CNRS, Chemistry of Materials Research Group LP3 UMR 7341, Marseille, F-13288, France; 2 Aix Marseille Université, CNRS, Electrochemistry of Materials Research Group MADIREL UMR 7246, Marseille, F-13397, France; 3 Aix Marseille Université, CNRS, CROPS ICR UMR 7273, Marseille, F-13397, France; 4 IPREM-ECP-UMR 5254, Université de Pau et des Pays de l'Adour, Hélioparc Pau-Pyrénées, 2 Av du Président Angot, Pau Cedex 9, 64053, France; 5 Aix Marseille Université, CNRS, CINaM UMR 7325, Marseille, F-13288, France

**Keywords:** Titania, Nanotubes, Electrodeposition, Copolymer electrolyte, Microbatteries

## Abstract

The highly conformal electrodeposition of a copolymer electrolyte (PMMA-PEO) into self-organized titania nanotubes (TiO_2_nt) is reported. The morphological analysis carried out by scanning electron microscopy and transmission electron microscopy evidenced the formation of a 3D nanostructure consisting of a copolymer-embedded TiO_2_nt. The thickness of the copolymer layer can be accurately controlled by monitoring the electropolymerization parameters. X-ray photoelectron spectroscopy measurements confirmed that bis(trifluoromethanesulfone)imide salt was successfully incorporated into the copolymer electrolyte during the deposition process. These results are crucial to fabricate a 3D Li-ion power source at the micrometer scale using TiO_2_nt as the negative electrode.

## Background

Nowadays, microbatteries are in demand as power source to drive small devices such as smartcards, medical implants, sensors, etc. To date, the electrochemical performances of these all-solid-state batteries are limited because planar thin films are employed as electrode and electrolyte materials. In general, the total thickness of the stacking films is below 15 μm, and the resulting battery reveals relatively low power and energy densities. In order to ensure significant advances for extended applications, it is crucial to improve the electrochemical performances by investigating new materials and manufacturing processes. In this context, the large specific area offered by nano-architectured electrodes represents a promising alternative to improve the general performances of these micro-power sources [1].

Particularly, better rate capability, capacity, and cycling behavior have been observed for self-organized nanostructures such as titania nanotubes (TiO_2_nt) [2-9]. However, when targeting 3D microbatteries, the conventional top-down approach to deposit solid electrolyte (e.g., lithium phosphorous oxynitride) [10-12] is not really suitable due to the accumulation of the electrolyte at the top of the nanotubes [13]. Certainly, with this accumulation of electrolyte, the 3D paradigm of microbatteries cannot be realized. Thus, investigating the deposition of polymer electrolytes into nanostructures by electrochemical techniques is a convenient way to ensure the desired filling of the nanostructures [14]. Indeed, electropolymerization is particularly powerful to control the deposition of different polymers into various porous materials [15-19]. Very recently, the use of electrodeposition to fill TiO_2_nt with a layer of poly(methyl methacrylate)-polyethylene oxide, i.e., PMMA-(PEO)_475_ has been reported [13,20]. We have demonstrated that this simple bottom-up approach is adequate to deposit a homogeneous copolymer layer into titania nanotubes while improving the electrochemical performance. However, conformal coating of the nanotubes by the polymer electrolyte is required to design a 3D microbattery. In this work, it is reported that the conformal deposition of a PMMA-PEO electrolyte into self-organized TiO_2_nt can be obtained by controlling the electrodeposition parameters. The morphology and the chemical composition of the resulting copolymer-embedded TiO_2_nt materials are characterized by scanning electron microscopy (SEM) and transmission electron microscopy (TEM). The incorporation of lithium bis(trifluoromethanesulfone)imide, so-called LiTFSI salt, into the electrodeposited polymer is studied by X-ray photoelectron spectroscopy (XPS).

## Methods

Synthesis of self-organized TiO_2_nt has been widely reported for a wide range of applications [21-24]. In the present work, TiO_2_nt layers were produced by the electrochemical anodization of Ti foils using the Modulab potentiostat from Solartron Analytical (Hampshire, UK). Before the anodization, Ti foils with the 99.6+ % purity and 0.125-mm thickness were cut into pieces with the desired dimensions and sonicated sequentially during 10 min in acetone, propanol, and methanol sequentially. After that, the foils were rinsed with deionized water and dried with compressed air. The anodization process was carried out in an electrochemical cell containing a solution of 1 M H_3_PO_4_, 1 M NaOH, and 0.4 wt.% of HF. The setup consisted of Ti foil as the working electrode, a Pt grid as the counter electrode, and a Hg/Hg_2_SO_4_, K_2_SO_4_ (saturated) (*E*=0.64V vs NHE) reference electrode. A constant voltage of 20 V was applied during 2 h. The material was rinsed with deionized water and dried with compressed air immediately after the anodization process.

Then, an aqueous electrolyte containing 0.035 M LiTFSI was introduced into the cell and purged with N_2_ gas for 10 min before adding 4 g of the MMA-(PEO)_475_ monomer provided by Sigma Aldrich (St. Louis, MO, USA). It can be noticed that no initiator was added into the solution. The copolymer-embedded TiO_2_nt was obtained by cyclic voltammetry (CV) using the as-prepared TiO_2_nt layers as the working electrode and a Pt grid as the counter electrode. The CV curves were carried out in the potential window ranging from −0.4 to −2.5 V vs Hg/Hg_2_SO_4_, K_2_SO_4_ (saturated) with the scan rate of 25 mV/s. The number of cycles was varied from 1 to 10 in order to observe the influence of cycle number on the polymer electrolyte layers. After electropolymerization, the samples were dried at room temperature to evaporate part of the residual water. The morphology of the copolymer-embedded TiO_2_nt was studied by SEM and TEM analyses using a JEOL 6320 F SEM and a JEOL 2010 F TEM (JEOL Ltd., Akishima, Tokyo, Japan). XPS measurements were carried out with a Kratos Axis Ultra spectrometer (Kratos Analytical Ltd., Manchester, UK), using focused monochromated Al Kα radiation (hν = 1,486.6 eV). The XPS spectrometer was directly connected to an argon dry box through a transfer chamber to avoid moisture/air exposure of the samples. The analyzed area of each sample was 300 μm × 700 μm. Peaks were recorded with constant pass energy of 20 eV. The pressure in the analysis chamber was around 5 × 10^−8^ Pa. Short acquisition time spectra were recorded before and after each normal experiment to check that the samples did not suffer from degradation under the X-ray beam during measurements. Peak assignments were made with respect to experimental reference compounds, namely bulk anatase and/or rutile TiO_2_. The binding energy scale was calibrated from hydrocarbon contamination using the C 1 s peak at 285.0 eV. Core peaks were analyzed using a non-linear Shirley-type background*.* The peak positions and areas were optimized by a weighted least-square fitting method using 70% Gaussian and 30% Lorentzian line shapes. Quantification was performed on the basis of Scofield's relative sensitivity factors.

## Results and discussion

**Figure 1:**
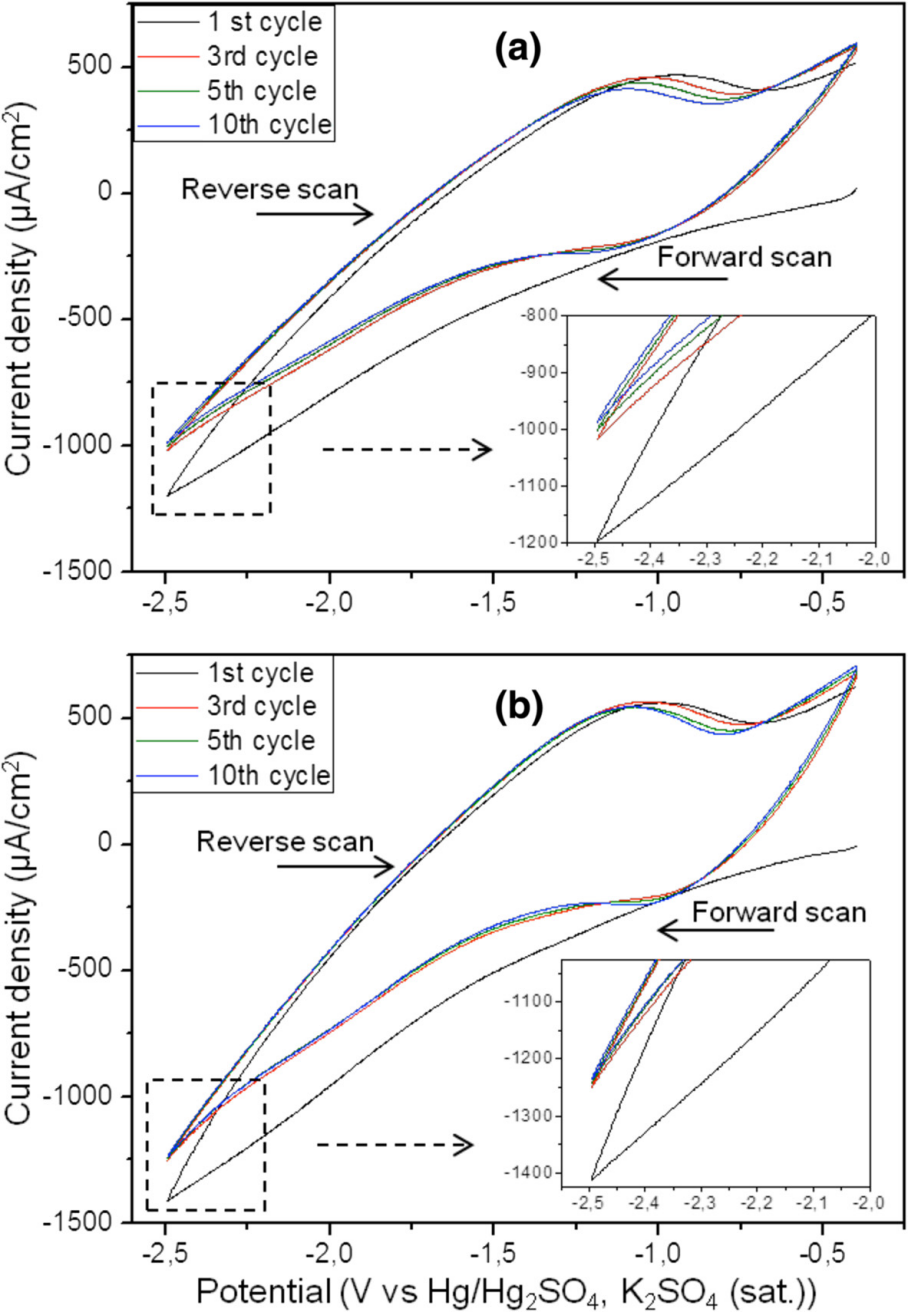
**Cyclic voltammograms of TiO**_**2**_**nt electrode.** In (**a**) 0.035 M LiTFSI and (**b**) in 0.035 M LiTFSI + 4 g MMA-PEO at the 1st, 3rd, 5th, and 10th cycles. The electropolymerization was carried out between −0.4 and −2.5 V vs Hg/Hg_2_SO_4_, K_2_SO_4_ (saturated) with the scan rate of 25 mV/s.

The electropolymerization of MMA-(PEO)_475_ monomer was carried out by cyclic voltammetry (CV) given in (Figure 1a). For comparison, the CV curves recorded in monomer-free electrolyte are given in Figure 1b. It is important to note that the monomer is not electrochemically active, and the polymerization process is expected to be initiated by free hydrogen radicals produced at applied cathodic potentials higher than −1 V vs Hg/Hg_2_SO_4_, K_2_SO_4_ (saturated) [13,20,25]. The similar shape of the CVs recorded in the two different electrolytes can be explained by the reactions involving the Ti^4+^/Ti^3+^ and H^+^/H_2_ redox couples. However, variations of the current densities obtained in the monomer-free electrolyte are almost independent of cycle number, suggesting that all electrochemical reactions are reversible without modification of the surface. In contrast, the cathodic current density decreases slightly with cycle number when the monomer is present. This effect on current density can be attributed to the thin copolymer layers successively deposited onto the titania nanotube walls and, consequently, acting as electrical insulators.

**Figure 2:**
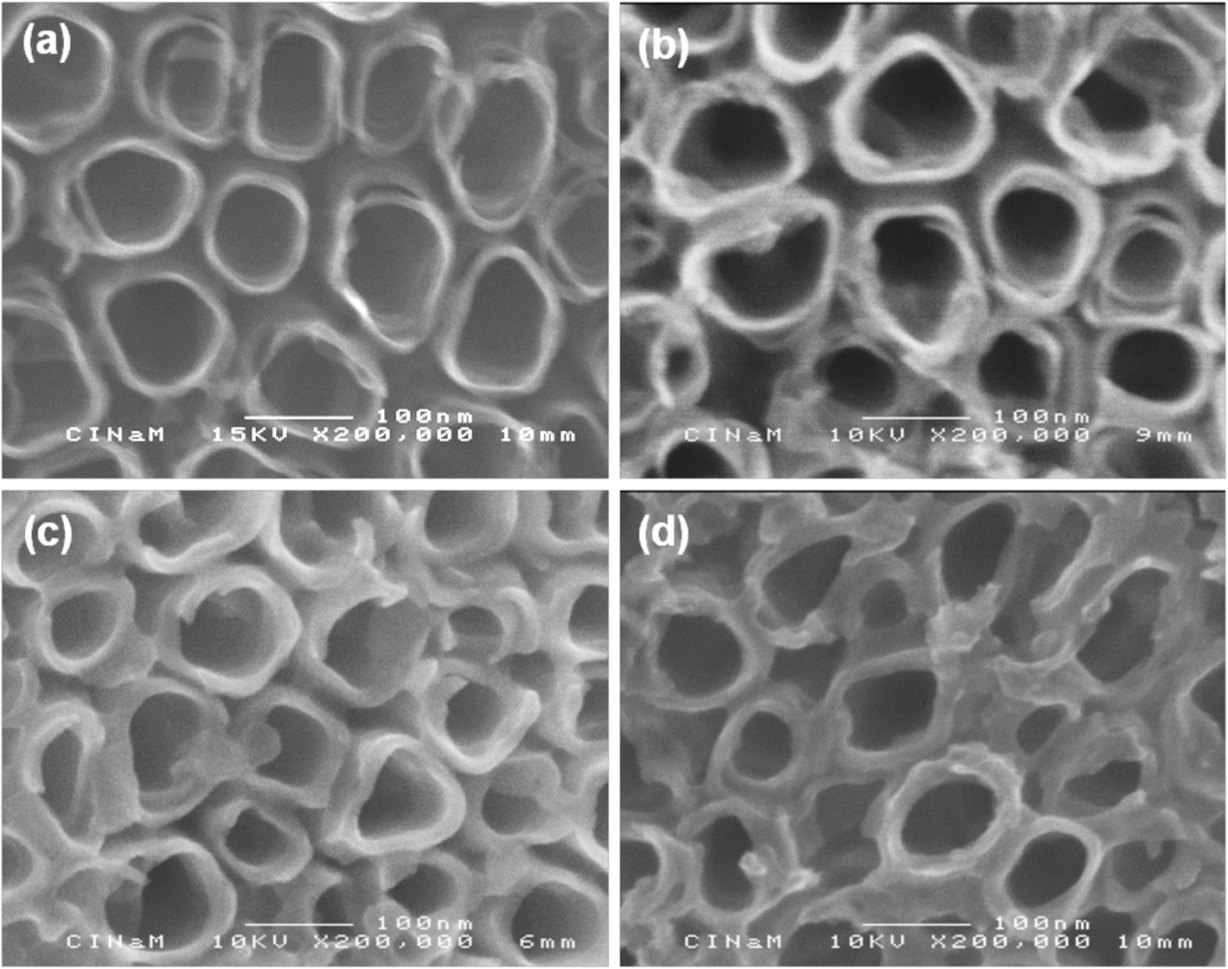
**SEM images.** (**a**) As-formed TiO_2_nt and (**b**) 1 cycle, (**c**) 5 cycles, and (**d**) 10 cycles copolymer-embedded TiO2nt.

The SEM images of the as-formed TiO_2_nt and the copolymer-embedded TiO_2_nt with different numbers of CV cycles are displayed in Figure 2. The evolution in thickness of the copolymer layer can clearly be observed. The SEM image of the as-formed TiO_2_nt shown in Figure 2a reveals very thin tube walls (around 9 nm) and presents some degree of perforation at the top. As the cycles of CV increase from 0 to 1, 5, and up to 10 according to Figure 2b,c,d, the average thickness of the nanotube walls increases from 9 to 13, 17, and 18 nm (see Table 1), respectively, while the inner diameters of the tubes and spaces between the tubes decrease from 90 to 84, 72 and 70 nm (Table 1), respectively. It can also be noticed that the perforation in the tube walls has clearly disappeared. These results confirm that the growth of a copolymer layer occurred on the inside and outside of each nanotube wall.

**Table 1:** Thickness of nanotube walls and inner diameters of tubes

**Number of cycles**	**Thickness of nanotube walls (nm)**	**Inner diameter of nanotubes (nm)**
0	9	90
1	13	84
5	17	72
10	18	70

**Figure 3:**
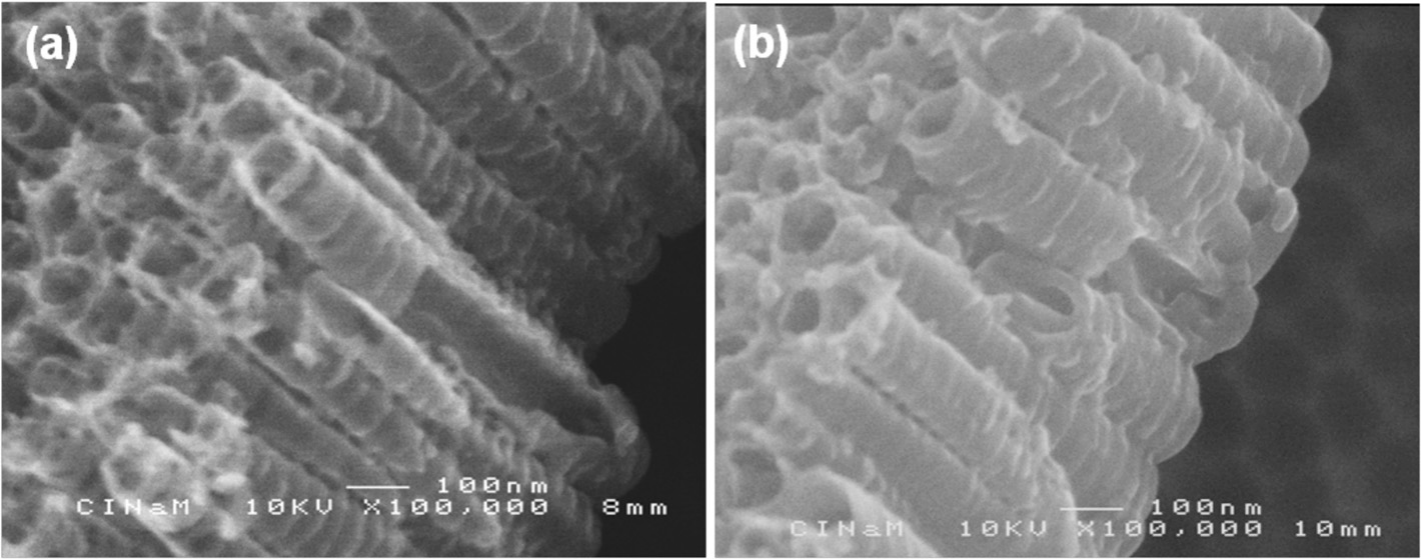
**Cross-sectional images of the copolymer-embedded TiO**_**2**_**nt after (a) 1 cycle and (b) 10 cycles.**

The deposition of the copolymer was further confirmed by examination of the cross-sectional SEM images given in Figure 3. Although the thin layer of copolymer is not obvious in cross-section after the first cycle (Figure 3a), it can easily be discerned along the nanotubes after 10 cycles of CV (Figure 3b). The inter-tube spaces are filled with the copolymer, and the nanotube walls become significantly thicker as compared to the sample obtained after 1 cycle of CV. It can also be noticed in these cross-sectional images (Figure 3a,b) that the perforations at the top of the tube are no longer visible owing to the filling and covering with the copolymer.

**Figure 4:**
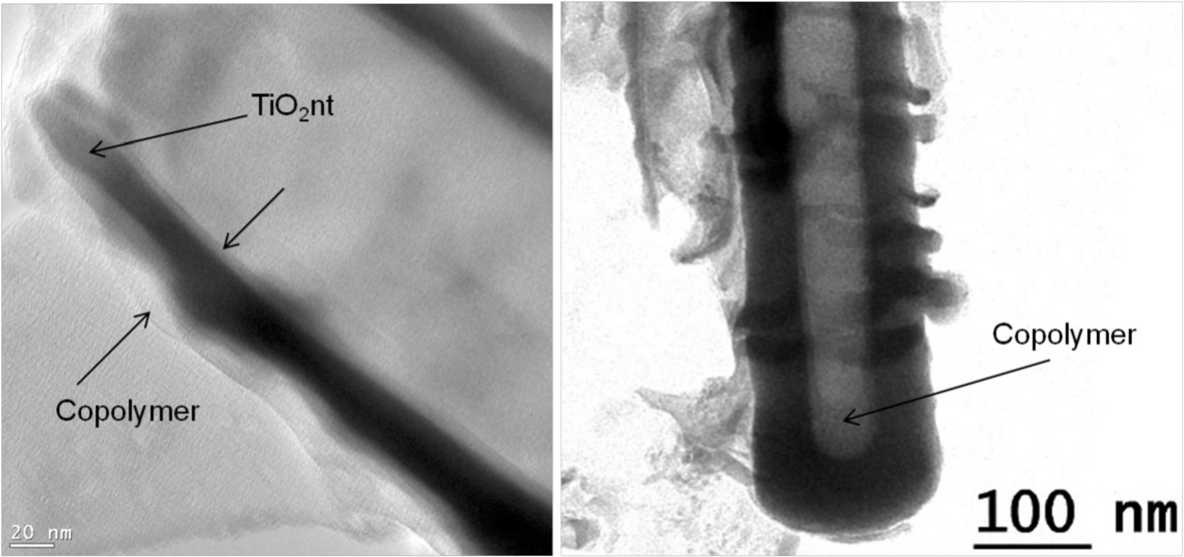
**TEM images of copolymer-embedded TiO**_**2**_**nt after 5 cycles of CV.** (**a**) Deposition of a homogeneous copolymer layer onto the inner and outer walls of the nanotube and (**b**) examination of the bottom part of the tube.

In order to verify the conformal deposition of the copolymer, TEM imaging was performed on a single nanotube. The TEM images of the TiO_2_nt after 5 cycles of CV are displayed in Figure 4. From Figure 4a, it can clearly be observed that a homogeneous copolymer layer of 6 nm in thickness has been deposited onto the inner and outer walls of the nanotube. This conformal coating along the nanotube wall can be confirmed by examining the bottom part of the tube (Figure 4b). A thicker deposit is observed at the bottom of the tube, suggesting that the copolymer initially grows from the bottom to the top of the tube.

**Figure 5:**
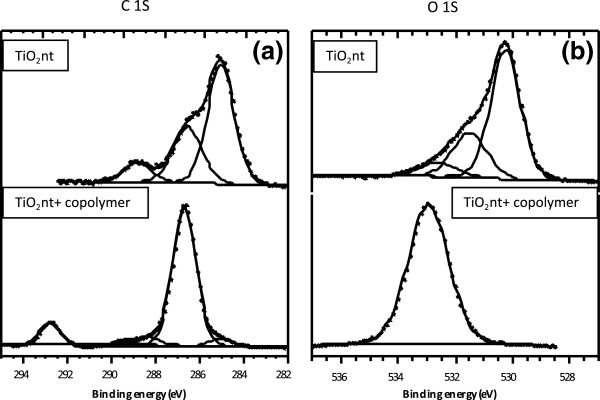
**XPS spectra.** (**a**) C 1 s and (**b**) O 1 s regions for as-formed TiO_2_nt and copolymer-embedded TiO_2_nt.

In order to confirm the presence of the copolymer and lithium salt (LiTFSI) in the self-organized TiO_2_nt structure, XPS measurement was performed. The XPS spectra of C 1 s and O 1 s binding energies of TiO_2_nt and copolymer-embedded TiO_2_nt are displayed in Figure 5. Concerning the C 1 s core peak (Figure 5a), the binding energy of C-H and C-O is clearly different around 285 and 286.5 eV. For copolymer-coated TiO_2_nt, the main contribution of this peak consists of a component located at 286.7 eV. This component is associated to the presence of oligomeric species of PEO (−CH_2_-CH_2_-O-)_*n*_ for which all carbon atoms are in a one-oxygen environment [26]. A sole peak at 292.8 eV can be assigned to CF_3_-like carbon atoms in LiTFSI (LiN(SO_2_CF_3_)_2_) as previously observed by Leroy et al. [27] in agreement with Ensling et al. [28]. The C 1 s peak for TiO_2_nt could be decomposed into three components respectively assigned to C-C (285.0 eV), CO (286.5 eV), and O-C = O (288.8 eV). For the O 1 s spectra (Figure 5b), two main peaks of as-formed TiO_2_ at 530.2 (Ti-O) and 531.5 eV (O-H hydroxyl) are absent in the copolymer-embedded TiO_2_nt sample. The absence of these peaks results from the coverage of TiO_2_nt with the copolymer. The broad peak of the copolymer-embedded TiO_2_nt sample may be assigned to O in PEO and LiTFSI.

**Figure 6:**
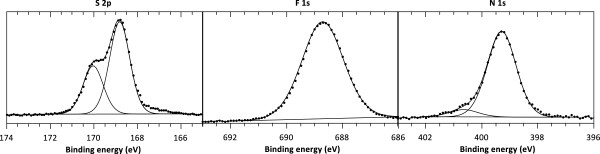
**XPS spectra of S 2p, F 1 s, and N 1 s regions of copolymer-embedded TiO**_**2**_**nt.**

The presence of LiTFSI is confirmed by the XPS spectra of the copolymer-embedded sample at S 2p, F 1 s and N 1 s binding energies as shown in Figure 6. According to Leroy et al. [27], the XPS core peak characterization of LiTFSI was achieved. In their work, the binding energy of N 1 s spectrum is located at 399.6 eV (399.4 eV in this work). The F 1 s, Li 1 s, and S 2p_3/2_ core peaks appear respectively at 688.6, 56.6, and 169.0 eV (688.7, 56.7, 168.8 eV in the present study). Concerning C 1 s spectrum, a sole peak at 293.0 eV can be observed for LiTFSI (LiN(SO_2_CF_3_)_2_), assigned to CF_3_-like carbon atoms. Here, the C 1 s core peak corresponding to C-F_3_-like carbon atoms is located at 292.8 eV. In conclusion, our results are in perfect agreement with those previously published. We can, thus, conclude to the evident presence of LiTFSI in the copolymer-embedded sample.

## Conclusions

The highly conformal electrodeposition of copolymer electrolyte has been successfully achieved on titania nanotubes. It is demonstrated that control of the electropolymerization parameters allows to homogeneously cover the nanostructures without closing the tubes. By this technique, the copolymer-embedded titania nanotubes retain the 3D structure which is advantageous for the further fabrication on high-performance 3D microbatteries.

## Competing interests

The authors declare that they have no competing interests.

## Authors' contributions

NP conducted the experiments and prepared the manuscript. NAK participated in the experiments. ME, FD, LS, and PK participated in the discussion. HM carried out the XPS studies. TD supervised the work. All authors read and approved the final manuscript.
